# P38α MAPK Signaling—A Robust Therapeutic Target for Rab5-Mediated Neurodegenerative Disease

**DOI:** 10.3390/ijms21155485

**Published:** 2020-07-31

**Authors:** Ursula A. Germann, John J. Alam

**Affiliations:** EIP Pharma, Inc., Boston, MA 02116, USA; ugermann@eippharma.com

**Keywords:** p38 MAPK, p38α, Rab5, endosome, Alzheimer’s, Lewy Bodies, amyloid-β, tau, α-synuclein

## Abstract

Multifactorial pathologies, involving one or more aggregated protein(s) and neuroinflammation are common in major neurodegenerative diseases, such as Alzheimer’s disease and dementia with Lewy bodies. This complexity of multiple pathogenic drivers is one potential explanation for the lack of success or, at best, the partial therapeutic effects, respectively, with approaches that have targeted one specific driver, e.g., amyloid-beta, in Alzheimer’s disease. Since the endosome-associated protein Rab5 appears to be a convergence point for many, if not all the most prominent pathogenic drivers, it has emerged as a major therapeutic target for neurodegenerative disease. Further, since the alpha isoform of p38 mitogen-activated protein kinase (p38α) is a major regulator of Rab5 activity and its effectors, a biology that is distinct from the classical nuclear targets of p38 signaling, brain-penetrant selective p38α kinase inhibitors provide the opportunity for significant therapeutic advances in neurogenerative disease through normalizing dysregulated Rab5 activity. In this review, we provide a brief summary of the role of Rab5 in the cell and its association with neurodegenerative disease pathogenesis. We then discuss the connection between Rab5 and p38α and summarize the evidence that through modulating Rab5 activity there are therapeutic opportunities in neurodegenerative diseases for p38α kinase inhibitors.

## 1. Introduction

Due to the scarcity of effective treatments for neurodegenerative diseases, urgent searches for candidate cellular and molecular mechanisms to develop therapeutic interventions are underway [1,2,3,4,5,6,7,8]. Neurodegenerative diseases, including Alzheimer’s disease (AD), Parkinson’s disease (PD), dementia with Lewy bodies (DLB), frontotemporal dementia (FTD), amyotrophic lateral sclerosis (ALS), and Huntington’s disease (HD) are typically defined by aberrant accumulations of one or more specific protein(s) and by loss of certain neuronal populations resulting in anatomic vulnerability. However, it is becoming increasingly clear that different neurodegenerative diseases exhibit common, central processes associated with progressive neuronal dysfunction and death, revealing multifactorial pathologies, including proteotoxic stress, neuroinflammation, and other abnormalities [3,5,6,9,10]. Even though the species of accumulating proteins are distinct in different neurodegenerative diseases, increasing evidence indicates that defects in the protein clearance system play a central role in the gradual accumulation of protein aggregates. Emerging genetic and biological evidence suggests that the endo-lysosomal protein degradation machinery, which is part of a unified pathway together with the autophagosomal machinery, is dysfunctional across a broad spectrum of neurodegenerative diseases, including AD, PD, ALS, HD, and others [1,11,12,13].

In this review we focus the discussion on the abnormal activity of the Ras-related protein Rab5, the master regulatory guanosine triphosphatase (GTPase) in early endosomes and highlight its role as a mediator of AD and other neurodegenerative diseases. We also discuss the relevance of Rab5 as a target that is affected by the p38α isoform of p38 mitogen-activated protein kinase (MAPK), hence, can be modulated by specific p38α inhibitors. The main objectives of this review are as follows: (1) we briefly review the role of p38α in the cell, including the neuron; (2) we review the role of Rab5 in the cell and discuss the association of dysregulated Rab5 activity with neurodegenerative disease pathogenesis; (3) we discuss the connection between Rab5 and p38α; (4) we provide evidence that through modulating Rab5 activity there are therapeutic opportunities in neurodegenerative diseases for brain-penetrant, selective p38α kinase inhibitors; and (5) we offer ideas for further investigations to increase the understanding of the mechanism of action of p38α kinase inhibitors on Rab5 in neurodegenerative disease.

## 2. Overview of the p38α Isoform as a Member of the p38 MAPK Family

The p38 MAPK family consists of four members that are encoded by separate genes and are known as p38α/MAPK14, p38β/MAPK11, p38γ/MAPK12/ERK-6/SAPK3, and p38δ/MAPK13/SAPK4 [14]. These four major isoforms differ in their organ, tissue, or cellular expression patterns, and it is becoming increasingly clear that they exert distinct biological functions [4,15,16,17,18,19,20,21,22,23]. Among the p38 MAPK family members, p38α was discovered first as a stress-activated protein kinase that plays a central role in inflammation [24,25]. P38α is also the best characterized isoform to date as a central nervous system drug discovery target [19,26,27].

In the adult mouse, p38α is highly expressed in different brain areas, including the cerebral cortex, hippocampus, cerebellum, and a few nuclei of the brainstem [28]. Neuronal cells are the predominant cell type expressing p38α [28]. At the subcellular level, p38α is distributed in dendrites and in cytoplasmic and nuclear regions of the cell body of neurons [28].

Many studies that have characterized p38α isoform function (frequently together with p38β function) have shown that it is an intracellular protein kinase involved in transducing intracellular (e.g., DNA damage) and extracellular (e.g., osmotic stress, infection) signals into a cellular response (e.g., inflammation or activation of other cellular stress responses) [17,29]. The major signal transduction pathway for p38α has been extensively studied and involves upstream activators (e.g., the mitogen-activated protein kinase kinases MKK6, MKK3) and downstream targets (e.g., mitogen-activated protein kinase activated kinase 2, also known as MAPKAPK2 or MK2) [17,29]. In the classic pathway for activating the proinflammatory response, some studies showed that inactive p38α (as well as p38β) is sequestered in the cytoplasm through its binding to MK2; upon activation, p38α phosphorylates MK2, leading to its dissociation [17,29]. Once dissociated, p38α translocates to the nucleus where it phosphorylates transcriptional machinery targets (e.g., histones, mitogen- and stress-activated kinases MSK1/2) in the proinflammatory context around nuclear factor NF-κB-associated targets [17,29]. In terms of therapeutics development, this understanding has led to a range of efforts to develop p38α kinase inhibitors (many of them primarily inhibiting p38α and p38β activity) as anti-inflammatory agents for chronic inflammatory disorders, including rheumatoid arthritis (RA), inflammatory bowel disease (IBD), and chronic obstructive pulmonary disease (COPD) [30,31].

However, p38α signaling has many targets aside from this classical pathway [32] and biologic effects other than regulation of proinflammatory cytokine production. With regard to neurodegenerative diseases, a large variety of biological roles have been attributed to p38α in brain pathology which depend on the type and stage of central nervous system (CNS) disease, brain region, cell type [4,18,19,26]. These roles include modulation of proinflammatory cytokine, e.g., interleukin-1beta (IL-1β) and tumor necrosis factor alpha (TNFα) production and signaling (e.g., in glia, microglia, astrocytes, neurons), as well as orchestration of neurotoxicity, neuroinflammation, and/or synaptic dysfunction, among others [4,18,19,26].

While the first study to characterize the role of p38α in regulating stress-induced endocytosis and early endosomal biology via modulating the activity of the endosomal protein Rab5 was published nearly two decades ago [33], this effect has not been a focus of intense follow-up research. Nevertheless, this biology, specifically within the neuron, has come to the forefront as potentially the most relevant for AD and other neurodegenerative disorders as will be discussed in Section 5.

During the last two decades, deregulated p38α has emerged as a leading therapeutic target for AD and has also been associated with the pathology of other neurodegenerative disorders, including PD, DLB, or ALS [4,19,34,35,36,37,38,39,40,41,42]. These therapeutic opportunities for treatment with selective, brain-penetrant p38α inhibitors will be discussed in more details in Section 6.

## 3. The Endosome-Associated Protein Rab5

Endocytosis represents the process to internalize diverse cargos (e.g., extracellular macromolecules, viruses, bacteria, membrane proteins) into cells (including neurons) through vesicles that bud off from the plasma membrane [43]. After their internalization into the cytosol, the endocytic vesicles are rapidly targeted to and fused with the early endosome [44,45,46]. This functions as the primary sorting organelle from which endocytosed cargo (e.g., select receptors) is either recycled back to the plasma membrane, or delivered to the lysosome/vacuole for degradation after maturation of the early endosome into a late endosome [44,45,46]. Important roles of early endosomes include nutrient uptake, degradation of metabolic by-products, transport of materials to specific compartments in the cell, and regulating the cell-surface expression of receptors and transporters [44,45,46].

### 3.1. Overview of Rab5 Roles

The Ras-related protein Rab5 is a small GTPase that is a major regulator of early steps of endocytosis, and subsequent endosomal membrane trafficking, sorting and endosomal fusion [47,48,49]. Further, through interacting with effector proteins Rab5 has a critical role in regulating the docking and fusion of endosomal membranes, endosomal mobility and intracellular signal transduction [48,50]. Rab5 effector protein include EEA1 (early endosomal autoantigen 1), APPL (adaptor protein, phosphotyrosine interacting with pleckstrin homology (PH) domain and leucine zipper 1), PI3K (phosphatidylinositol-3-kinase), Rabenosyn-5/hVPS45 (human Sec1p-like vacuolar protein sorting), or Rabaptin-5/Rabex-5, among others. Additionally, a role of Rab5 in regulating the internalization and trafficking of membrane receptors by regulating vesicle fusion and receptor sorting in the early endosomes is emerging [49]. Rab5, which actually comprises three different isoforms, is among the best characterized endosomal markers, in part because of its abundant expression and ubiquitous tissue distribution, including neurons [51,52].

### 3.2. Rab5 Importance for Neuronal Function

It is clear that proper Rab GTPase function is critical for normal (wild-type) neuronal function, including trafficking for pre- and post-synaptic function as well as dendritic trafficking [52,53]. Studies in *Drosophila* have demonstrated that Rab5 is required for synaptic endosomal integrity, synaptic vesicle exo-/endocytosis rates, and neurotransmitter probability [54]. Furthermore, an essential function is that Rab5-dependent endosomal sorting may regulate the uniformity of synaptic vesicle size [55].

The neuron may be particularly sensitive to dysregulation of Rab5 activity for at least two main reasons: (1) Endocytosis and subsequent recycling (or not) regulate the concentration of neurotransmitter receptor density on the cell surface, determining signal strength [53,56,57]. For example, α-amino-3-hydroxy-5-methyl-4-isoxazolepropionic acid receptor (AMPAR) endocytosis in hippocampal neurons leads to long-term depression (LTD), and Rab5 is essential in this process [56,58,59]; and (2) neurotrophin signaling from synapses is dependent on endocytosis, retrograde transport of endosomes along axons, and endosomal signaling [1,48,52,60].

### 3.3. Rab5 Therapeutic Targeting Strategies

The activity of Rab5 is coordinately regulated and, therefore, can be therapeutically targeted at several levels through modulation of Rab5 regulatory proteins. Firstly, Rab5 is shuttled between membranes by the general Rab regulator GDP dissociation inhibitor (GDI) [61]. This serves to release Rab5 that is bound to GDP, Rab5(GDP), from membranes to maintain Rab5 in the cytoplasm, and to recycle it back to donor membranes [61]. Thus, factors that increase formation of the Rab5-GDI complex also increase delivery of Rab5 to the plasma membrane where it can act [61]. Secondly, at the membrane, the activity of Rab5 is regulated by guanine nucleotide exchange factors (GEFs) and GTPase-activating proteins (GAPs) that determine the proportion of Rab5 bound to either GDP (Rab5(GDP); inactive state) or GTP (Rab5(GTP); active state) [62]. Thirdly, Rab5 activity is modulated by other factors that impact the effectors; for example, the phosphorylation of, and activity of, PI3K or EEA1 [63,64,65,66]. Additionally, the druggability of membrane-bound Rab5 itself, the selective inhibition of Rab5 GTPase activity, or blocking membrane recruitment through inhibition of Rab5 prenylation, or targeting Rab5-associated signaling pathways can be explored [67,68,69].

## 4. Role of Dysregulated Rab5 in the Pathogenesis of Neurodegenerative Disease

Dysregulated Rab5 activity has been defined as a major pathogenic driver in AD [1,48,70]. Moreover, a pathogenic role of aberrant Rab5 is emerging in many of the same other neurodegenerative diseases that are being targeted by p38α inhibitor programs, including PD, DLB, ALS, and HD [71,72,73]. Rab5 is a member of a large family of Rab proteins involved with neuronal function [53,71] and a number of other Rab proteins have been connected to neurodegenerative disease. However, as will be discussed in Section 5, Rab5 activity has been robustly connected to p38 MAPK signaling, while no such connection has been established for the other Rab proteins. Therefore, this review is focused on Rab5, and the reader is referred to a number of other excellent recent reviews on the broader family of Rab proteins and their relation to the pathogenesis of neurodegenerative disease [71,72,73].

### 4.1. Dysregulated Rab5 as Therapeutic Target in AD

Neuronal endocytic pathway activation is a specific and very early response in AD that precedes amyloid-beta (Aβ) deposition in sporadic AD, hence, the role of dysregulated Rab5 in AD has been extensively studied and reviewed elsewhere [1,48,70,71]. It will be discussed briefly here.

In a large series of experiments during more than two decades, Nixon and colleagues have documented specific impairments of the endosomal-lysosomal system at the earliest stage of AD and linked the genetic drivers that cause AD directly to functions within endocytic and autophagic pathways of the lysosomal system. They demonstrated that abnormal Rab5-positive endosome enlargement is the earliest pathologic event in sporadic AD patients [74,75]. They also showed that abnormal Rab5-positive endosome enlargement is the earliest pathologic event in Down syndrome (DS) patients [74,75]. DS patients are individuals with trisomy for all or part of third copy of chromosome 21 (which carries the β-Amyloid Precursor Protein (APP) gene among others), who nearly uniformly develop progressive AD after age 40 [74,75]. Importantly, Nixon and colleagues also defined the mechanistic basis of the endosome enlargement induced by APP to be Rab5 hyperactivation [70]. They also linked functional neuronal deficits and, where evident, subsequent neuronal loss in animal models of AD and DS to Rab5 hyperactivation [70].

Among other lines of evidence, Nixon and colleagues showed that the β-cleaved carboxy-terminal fragment of APP, termed β-CTF, recruits APPL1 to Rab5 endosomes [76]. There APPL1 stabilizes active Rab5(GTP), leading to pathologically accelerated endocytosis, endosome swelling and selectively impaired axonal transport of Rab5 endosomes [76]. Importantly, in DS fibroblasts an APPL1 knockdown corrected these endosomal anomalies [76]. β-CTF levels were also shown to be elevated in AD brain, which was accompanied by abnormally high recruitment of APPL1 to Rab5 endosomes, as was observed in DS fibroblasts [76]. Moreover, in a separate report, Nixon and colleagues [77] showed that partial reduction of β-APP cleaving enzyme 1 (BACE1) through genetic means in a transgenic mouse model (Ts2) of DS normalized both APP-β-CTF levels and Rab5 activation. This prevented age-related development of Rab5-positive endosomal enlargement (which is usually evident at approximately four months of age in the Ts2 mice) and subsequent loss of cholinergic neurons in the basal forebrain (which otherwise follows the Rab5-positive endosomal enlargement within approximately one to two months in the Ts2 mice) [77].

In complementary work, Xu et al. [78] demonstrated that full-length wild-type APP and β-CTF both, in vitro in three different relevant cell model systems, induced early endosomes enlargement and disrupted nerve growth factor (NGF) signaling and axonal trafficking. Moreover, β-CTF alone induced atrophy of cultured rat basal forebrain cholinergic neurons that was rescued by a dominant-negative Rab5 mutant [78]. Finally, expression of a dominant negative Rab5 construct markedly reduced APP-induced axonal blockage in *Drosophila* [78].

This earlier work indicated that Rab5 was necessary for APP-induced endosomal enlargement and cholinergic neuronal loss. Recently, Nixon and colleagues demonstrated that abnormal Rab5 activation is sufficient to induce endosomal enlargement and a neurodegenerative phenotype which mimics that seen with APP overexpression [79]. Specifically, modest neuron-specific transgenic Rab5 (PA-Rab5) expression in mice [79] induced increased Rab5 expression and abnormal activation of Rab5 comparable to that in AD brain [80,81]. PA-Rab5 reproduced AD-like Rab5-endosomal enlargement and mis-trafficking without impacting APP metabolism (i.e., no increase in Aβ levels) [79]. The PA-Rab5 mice also exhibited hippocampal synaptic plasticity deficits via accelerated AMPAR endocytosis and dendritic spine loss [79]. Moreover, they showed tau hyperphosphorylation [79]. Importantly, with further aging the PA-Rab5 mice developed progressive cholinergic neurodegeneration and impaired hippocampal-dependent memory subsequent to the observed Rab5-mediated endosomal dysfunction [79].

That Rab5 hyperactivity and endosome enlargement, rather than APP per se, are the critical factors in inducing degenerative AD-related changes is further supported by the following findings. Age-dependent Rab5-positive early endosome enlargement and endo-lysosomal dysfunction were observed in an AD-vulnerable brain region of targeted replacement mice expressing the human Apolipoprotein E4 (ApoE4) gene, the dominant genetic factor for the development of late-onset Alzheimer’s disease, under the control of the endogenous murine promoter [82]. Similarly, depletion of another late onset AD risk gene sortilin-related receptor 1 (SORL1) [83] in human induced pluripotent stem cell (iPSC)-derived neurons leads to enlargement of early endosomes (i.e., endosomes staining positive for Rab5 and EEA1). Hence, genetic influences that increase AD risk, such as ApoE4 and SORL1, may do so by dysregulating the Rab5 impact on endosome dynamics and cell signaling.

A recent study that analyzed a comprehensive panel of iPSC-derived neuronal lines relevant to familial AD also demonstrated translatability of Rab5-mediated neurodegeneration to human AD [84]. The only consistent, intra-neuronal physiologic defect identified was enlargement of Rab5-positive early endosomes, mediated by APP-β-CTFs, not Aβ, and associated with endosomal/endocytic dysfunction [84].

**Figure 1:**
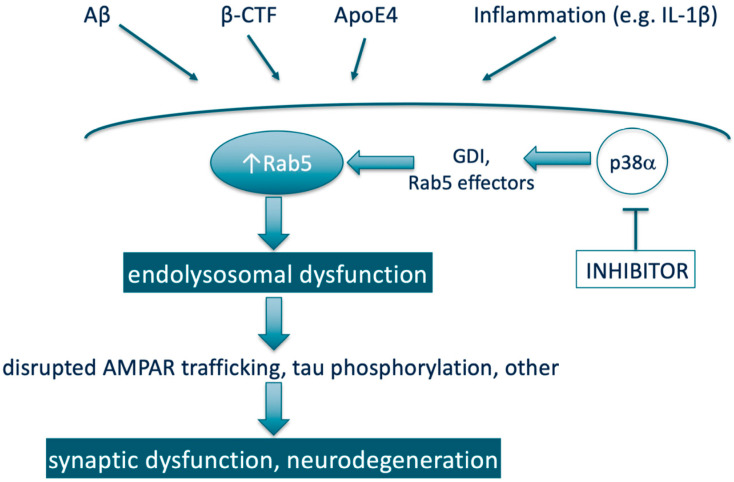
Simplified scheme representing Rab5 as a therapeutic target through being a convergence point for multiple pathogenic drivers of neurodegeneration in AD and the potential of p38α inhibition as a therapeutic lever to reduce Rab5 activity. Note: Aβ and β-CTF are both derived from proteolytic processing of APP.

In a very simplified view of many AD research data taken together, the small GTPase Rab5 can be depicted as a convergence point (Figure 1) for multiple established pathogenic drivers of neurodegeneration in AD (e.g., downstream of APP, APP-β-CTF, β-CTF, Aβ, ApoE4, and others). Dysregulation of the endo-lysosomal system represents the important early cellular phenotype of pathogenesis for AD that leads to disrupted AMPAR trafficking, tau pathology, synaptic dysfunction, and neurodegeneration. Elevated Rab5 activity (hyperactivation or overexpression) plays the key role in mediating these processes, hence, may promise high potential as a therapeutic target. Since endosome dysfunction occurs very early in the AD pathogenic process, therapies that turn Rab5 activity back to normal (e.g., via Rab5 therapeutic targeting strategies already discussed in Section 3.3.) may help slow or halt AD development before irreversible damage occurs. Additionally, the potential of p38α inhibition as a therapeutic lever to reduce Rab5 activity will be discussed in Section 6.

### 4.2. Dysregulated Rab5 Associated with Abnormal α-Synuclein in PD, DLB, and AD

The enlargement of Rab5-positive early endosomes that is seen in AD is not observed during the development of PD and DLB. Nevertheless, the toxicity of the key pathologic protein associated with these two neurodegenerative diseases, abnormal α-synuclein (a pre-synaptic protein), has also been linked to Rab5 [71,85,86]. Specifically, the neurotoxicity of α-synuclein was shown to be dependent on Rab5-mediated entry into the cell via endocytosis [87,88]. Expression of a GTPase-deficient Rab5a protein led to a decrease in the cytotoxicity of α-synuclein through impairing its endocytosis [87]. Rab5 also appears to play a role in intracellular trafficking of α-synuclein [89,90]. Additionally, studying embryonic cortical neurons from a mouse model of Parkinson’s disease, transgenic overexpression of α-synuclein was observed to increase the levels of activated Rab5 and Rab7 [91]. This impaired retrograde axonal transport of brain-derived neurotrophic factor (BDNF) and led to neuronal atrophy [91]. Therefore, the authors suggested that α-synuclein-induced neuronal dysfunction is a result of impaired endocytosis and endosomal dysfunction associated with aberrant activation of the two Rab proteins [91].

It is interesting to note that accumulating evidence suggests that the α-synuclein might also play a role as driver of pathophysiology in AD [92]. Intriguingly, α-synuclein and APP appear to be interconnected in terms of their activation of Rab5 and neurotoxicity, since genetic reduction of endogenous α-synuclein in an APP transgenic mouse model normalized Rab5 (and Rab3) activity and prevented cholinergic neuronal loss [93].

### 4.3. Dysregulated Rab5 in ALS

Evidence for the pathological role of Rab proteins has also been provided in ALS as another example of a neurodegenerative disease involving endosomal-lysosomal trafficking and signaling defects [71].

In the context of ALS, defects in endosomal trafficking have been consistently seen in transgenic mouse models based on identified human genetic defects [94]. In particular, Alsin, deficiency of which is associated with an autosomal recessive juvenile form of ALS called ALS 2, is a Rab5 exchange factor [95,96,97]. The primary biological effects of Alsin deficiency have been linked to aberrant activation of Rab5-mediated endosomal trafficking [98]. Rab5 interaction with Alsin has also been suggested to modulate the signaling of neurotrophic factors [96]. The analysis of Alsin-null mice, an animal model of ALS2, revealed that Rab5-dependent endosome fusion activity and endosomal transport of insulin-like growth factor 1 (IGF1) and BDNF receptors were affected [99]. It was suggested that these alterations in trophic receptor trafficking in the neurons of the Alsin-null mice may lead to the observed reduced size of the cortical neurons as well as animal hypoactivity, and that this may translate to the pathogenesis of ALS2 [99].

Moreover, the protein product of hexanucleotide GGGGCC repeat in the chromosome 9 open reading frame 72 (C9ORF72), which represents a major genetic cause of familial ALS (33% of familial cases) and FTD, has been co-localized with Rab5 in endosomes [100,101]. It was described to possess Rab GEF activity and function as a regulator of endosomal trafficking [100].

### 4.4. Dysregulated Rab5 in HD

Finally, Rab proteins also have a key role in HD [52,71]. It was reported that the upregulated Huntingtin (Htt)-associated protein 40 (HAP40) is an effector of Rab5 that mediates the recruitment of Htt to early endosomes and is affecting early endosomal motility [102]. As Rab5-positive endosomes are involved in retrograde transport of activated neurotrophin/receptor complexes and due to indication of altered axonal transport in HD [103,104], it is possible that impaired Rab5-mediated trafficking of neurotrophins affects neurotrophin signaling and might also contribute to HD pathogenesis [105,106]. Moreover, Rab5 overexpression reduces toxicity of the Htt mutant protein, while inhibition of Rab5 increases toxicity via macroautophagy regulation [107].

Taken together, overactivated Rab5 and subsequent endo-lysosomal dysfunction have emerged as a major driving force of degenerative and cognitive deficits during the development of AD [1,48,71,79] and alterations in Rab5 also seem to play an important role in other types of neurodegenerative diseases [52,71].

## 5. p38α Is a Major Regulator of Rab5 Activity

It is well-established in the scientific literature that p38α regulates Rab5 activity. This includes the research in the context of neuronal synaptic plasticity. Most of the findings were published in the early and mid-2000s. First, p38α was shown to be a regulator of endocytosis through phosphorylating GDI and stimulating the formation of cytosolic Rab5-GDI complex, thereby increasing the concentration of Rab5 in the plasma membrane (Figure 1) [33,108]. Moreover, in a genome-wide screen of human kinase-mediated regulation of endocytosis, ablation of a number of kinases increased endocytosis in association with increasing phosphorylation of p38 MAPK (i.e., activated p38 MAPK) and recruiting phospho-p38α to the endosome [109]. These authors also showed by confocal microscopy that the MAPK14 gene product was observed on endosomal structures [109]. Prior to this, p38 had been co-localized via a sucrose gradient with the Rab5- and NGF-containing early endosome fraction prepared from rat dorsal root ganglion (DRG) neurons, and was shown to be part of early endosome signaling pathways for conveying NGF signals from the target of nociceptive neurons to their cell bodies [110]. In addition, expression of an activated Rab5 mutant increased µ opioid receptor endocytosis in wild-type cells but not in p38α -/- cells [111]. In the same report, p38α was also shown to phosphorylate the Rab5 effectors EEA1 (on Thr-1392) and Rabenosyn-5 (on Ser-215), which led to increased recruitment of these proteins to membranes; providing a mechanism other than modulating GDI by which p38α increases Rab5 action. Both in the human kinase screen [109] and the μ opioid receptor endocytosis studies [111] it is noted that the effects of p38α on endocytosis are evident under basal (physiologic) conditions, and not just under conditions of cellular stress, whereas the role of p38α in relation to endocytosis has been suggested to be related to its role in responding to oxidative stress [112]. Collectively, the studies indicate that p38α regulates levels of both the basal and induced Rab5 activity, irrespective of other inputs to Rab5 activation state. As such, p38α inhibition provides an approach to reduce Rab5 activity in a diverse range of disease states that may have different drivers of Rab5 activation (Figure 1).

In the context of neuronal function, a critical component of synaptic plasticity is the maintenance and/or recycling of AMPAR from the surface of synapses, [59] a process in which Rab proteins, including and particularly Rab5, through increasing endocytosis play a prominent role (Figure 1) [58]. In particular for the aspect of synaptic plasticity termed LTD, p38 MAPK activation facilitating AMPAR removal through increasing endocytosis via the Rab5-GDI complex has been demonstrated [56].

In other studies, in which Rab5 activation leading to AMPAR removal from the surface was thought be a critical player in the process of NMDA-triggered LTD induction in the hippocampus, there was associated phosphorylation of p38 MAPK, i.e., p38 MAPK activation; though there was a temporal lag, which may reflect different kinetics of p38 MAPK activation at the plasma membrane versus the cell as a whole [58]. Serotonin-induced LTD has also been shown to be dependent on both p38 MAPK and Rab5, activation of which together led to enhanced AMPAR internalization via endocytosis during the process of LTD [113]. In a subsequent report [114] the same group demonstrated low dose serotonin and norepinephrine reuptake inhibitors (SNRIs), by acting on 5-HT1A and 2-adrenergic receptors, synergistically reduced AMPAR-mediated excitatory postsynaptic currents and AMPAR surface expression in prefrontal cortex pyramidal neurons via a mechanism involving Rab5/dynamin-mediated endocytosis of AMPAR. As this effect of SNRIs was dependent on p38 kinase activity, and their prior work, they hypothesized that SNRI activation of p38 MAPK accelerates AMPAR endocytosis by stimulating the formation of Rab5-GDI complex. However, they did not directly demonstrate this.

## 6. Therapeutic Potential of Dampening Rab5 Activity through Inhibiting p38α Signaling

In parallel with Rab5 emerging as a therapeutic target for neurodegenerative disease, p38α has also emerged as a promising therapeutic target for AD and other neurodegenerative disorders [4,19,34,35,36,37,38,39,40,41,42].

### 6.1. Therapeutic Potential in AD

From a mechanistic perspective, expression of p38α in the neuron is associated with formation of pathological Aβ-, inflammation- (e.g., IL-1β) and tau-induced impaired synaptic plasticity (Figure 1), as well dendritic spine loss [115,116,117,118,119]. Furthermore, studies in several distinct animal models driven by Aβ, inflammation, or tau showed that spatial learning and working memory deficits are reversed with small molecule inhibitors of p38α kinase activity [120,121,122], providing direct evidence that inhibition of p38α activity has therapeutic potential in AD. Specifically, the compound MW150 was active in APP-transgenic and tau-transgenic mice [121,123], the compounds MW181 and SB2399063 in aged tauopathy mice [122], and neflamapimod/VX-745 in aged rats [120]. In addition, a very recent publication demonstrated that oral administration of a selective p38 α/β inhibitor, NJK14047, to 9-month old 5XFAD (APP) transgenic mice reduced levels of amyloid-beta deposits, reduced spatial memory loss and reduced the number of degenerating neurons labeled with Fluoro-Jade B [41]. Moreover, genetic reduction of neuronal p38α in APP overexpressing transgenic mice improved synaptic transmission, decreased memory loss and reduced amyloid pathology [124,125]. P38 MAPK has also been identified as a therapeutic target for PD and DLB, i.e., α-synuclein mediated neurodegenerative diseases [27,39].

Since none of the published studies assessed Rab5 activity and/or endosomal pathology at present, the literature does not definitively establish that the aformentioned effects of p38α in animal models of neurodegenerative disease are via modulating Rab5 activity. However, several arguments suggest that a major component of the therapeutic effects of p38α is through targeting Rab5. First, in the AD context AMPAR removal is necessary and sufficient for both impaired synaptic plasticity and dendritic spine loss [126], the critical first steps in the neurodegenerative process associated with AD; and as discussed previously, p38α and Rab5 are intimately linked in the process of AMPAR endocytosis and removal from the cell surface. Second, across the variety of biological effects of modulating either Rab5 or p38α activity there is a similarity of effects (including directionality) that, given the known connection between the two, is unlikely to be due to chance. For example, decreasing p38α activity in neurons reduces Aβ production [125], while Rab5 activation increases Aβ production [127]. That is, aberrant activation of either p38 MAPK [115,128] or Rab5 [79] are associated with increased Aβ production. Further, aberrant activation of Rab5 resulting in a block in endosomal maturation is considered to underlie impaired autophagy in AD [1], while inhibition of p38α has been identified as an approach to reversing impaired autophagy in AD [11]. Third, in the Rab5-overexpressing mouse a downstream biological marker in the neuron of Rab5 activation is tau phosphorylation [79], while p38α inhibitors in aged tauopathy mice improved working memory and, at same time, reduced tau phosphorylation [122]. As p38α is not a major tau kinase [129], we believe that those results provide indirect evidence that p38α inhibition reduces Rab5 activity in parallel with improving memory.

Recently [42], the effects of neuronal deficiency of p38α in neurodegenerative disease models were further evaluated by mating human APP transgenic mice and human P301S Tau-transgenic mice with *mapk14*-(gene for p38α)-floxed and neuron-specific Cre-knock-in mice. Deletion of p38α in neurons through this approach led to improvement of cognition in both the APP transgenic mice and the P301S Tau transgenic mice, associated with decreased Aβ and phosphorylated tau in the brain of the respective models. As along with normal Rab5 adequate calcium influx is essential to, and intimately associated with AMPAR endocytosis [130,131], it is particularly intriguing that neuronal deficiency of p38α in these models regulated the transcription of calcium homeostasis genes and deletion of p38α inhibited NMDA-triggered calcium influx in vitro [42].

More direct evidence on the contribution of Rab5 inhibition towards therapeutic effects of p38α inhibition have been presented at scientific meetings but are not available, yet, as primary research publications. In those studies [132,133] we and our collaborators showed that blocking Rab5 over-activation with a selective p38α inhibitor [37,134] rescued Rab5-positive-endosomal enlargement and cholinergic neurodegeneration in a mouse model of DS (Ts2) as effectively as reversing elevated APP-β-CTF levels [77]. The results directly support the role of p38α in regulating Rab5, as the compound utilized had previously been shown by an independent academic research group to have ~25-fold selectivity for p38α (Kd = 2.8 nM) versus p38β (Kd = 74 nM), as well as its >300-fold selectivity versus 445 other kinases (Kd ≥ 1100 nM) [135]. In addition, the compound had been recommended by yet another research group as the small molecule compound to utilize in experimental studies that have the objective of understanding the biologic effects of inhibiting p38α kinase activity [136].

### 6.2. Therapeutic Potential in ALS

In the context of ALS, it should also be noted that overexpression of the Rab5 GEF Alsin suppresses superoxide dismutase 1 (SOD1) neurotoxicity [137]. The link to p38α was recently established in studies that showed that p38α kinase inhibitors rescued the axonal transport defects in the SOD1^G93A^ mouse model of ALS [40]. In those studies, p38 MAPKs were found to enhance axonal transport of signaling endosomes in a pharmacological screen of a library of small molecule kinase inhibitors that was designed to identify molecules that would enhance that activity. Moreover, in vitro knockdown revealed that the alpha isoform (i.e., p38α) was the sole isoform responsible for the SOD1^G93A^-induced transport deficits and acute treatment with p38α inhibitors restored the physiological rate of axonal retrograde transport in vivo in early symptomatic SOD1^G93A^ mice [40].

## 7. Future Directions for Research and Prospects

**Figure 2:**
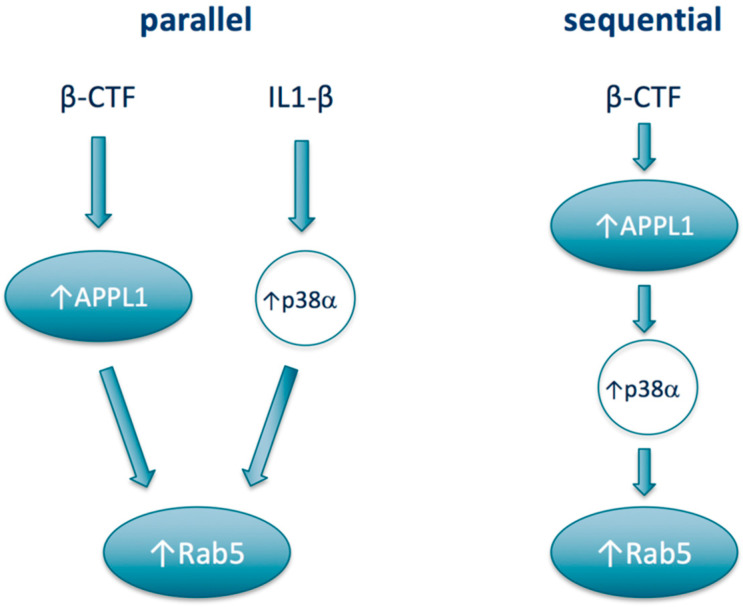
Potential models of relationships between APPL1, Rab5, and p38α.

From a therapeutics development perspective, the most relevant preclinical mechanistic study to assess the potential of p38α inhibition as a treatment approach for Rab5-mediated neurodegenerative disease would be to evaluate the effects of a p38α inhibitor in the Rab5-overexpressing mouse. Such studies have recently been initiated and the results are anticipated to be published within the next 12 months [138]. From a mechanistic standpoint, a number of open lines of inquiry around the connection between p38α and Rab5 could be explored. For one, in the AD context, whether APPL1 and p38α may act sequentially or in parallel to increase Rab5 activation has not been defined (Figure 2). On one hand, APPL1 as a scaffolding protein could stabilize active Rab5 on the endosome, while p38α activation leading to increased levels of Rab5-GDI complex would deliver Rab5 to the endosome to associate with APPL1. In this “parallel” construct, APPL1 and p38α could have different upstream drivers; for example, p38α being activated upstream by a well-known activator IL-1β, rather than by β-CTF. However, APPL1 has also been shown to act as a scaffold to the p38MAPK signaling pathway [139] and so may act also upstream of p38α, i.e., sequentially, rather than in parallel to activate Rab5. From a therapeutics development model, the two models could impact the context, e.g., the disease in which p38α inhibitors would be most active.

Another open question is the specific mechanism by which Rab5 activation leads to defects in endosomal signaling and trafficking, an effect that is, to a certain extent, paradoxical as increasing endocytosis and endosome formation would be expected to increase endosomal signaling and potentially increase the number of endosomes delivered from the synapse back to the nucleus via axonal transport. While some specific mechanisms have been proposed [1], the more general hypothesis is that axonal transport and endosomal degradation via lysosomal pathways are rate-limiting and have to be well-matched to the rate of endocytosis. As a result, aberrantly-increased endocytosis overwhelms the rate-limiting disposal pathways, leading to a block in trafficking/degradation and endosomal enlargement. While compelling, this hypothesis has not been definitely established as the reason for the reduction in axonal transport of endosomes that is seen in AD. Further understanding of these mechanisms might identify additional therapeutic targets.

More generally, with respect to p38 MAPK signaling, the roles that regulation of endocytosis and endosomal biology play in the stress response that is otherwise mediated by p38α, or other p38 MAPK isoforms, remain to be fully defined. With respect to the proinflammatory activity of p38α, along with increasing cytokine production, activation of p38α increases cytokine signaling. Classically receptor endocytosis is thought to shut off the signal from the receptor. However, there are increasing examples, including in the context of cytokine signaling, that receptor endocytosis can increase signaling [140,141,142]. Further, in the context of the neuron endosomal signaling after axonal retrograde signaling, both the signaling pathways distinct from neurotrophins [60] and the cross-talk on the endosome between kinase pathways [143] are underexplored in terms of understanding their roles in modulating p38α (or p38 MAPK) signaling.

The ultimate proof of the therapeutic value of targeting Rab5 with p38α will be in the clinic. Towards that end, results were presented recently [144] from a 24-week 161 patient double-blind, placebo controlled clinical trial of a p38α inhibitor neflamapimod in early-stage AD (https://clinicaltrials.gov/ct2/show/NCT03402659). This study demonstrated the effectiveness of p38α inhibition relative to a placebo in significantly reducing cerebrospinal fluid (CSF) levels of p-tau and tau. Given that as discussed previously tau phosphorylation is a downstream marker of Rab5 hyperactivation, the results provide indirect evidence that p38α inhibition impacts Rab5 activity in humans. The study also showed plasma concentration-dependent effects on episodic memory function, though the dose level utilized led to sub-therapeutic levels in the majority of the patients. A higher dose regimen that achieves the identified therapeutic plasma drug concentration range in ~75% if patients is being utilized in a 16-week randomized, double-blind, placebo-controlled, clinical study of the same p38α inhibitor in patients exhibiting dementia with Lewy bodies (https://clinicaltrials.gov/ct2/show/NCT04001517). The results of this study should further inform on the potential of p38α inhibition as an approach to treat neurodegenerative disease.

## Abbreviations

Aβ	Amyloid-β
AD	Alzheimer’s disease
ALS	Amyotrophic lateral sclerosis
AMPAR	α-Amino-3-hydroxy-5-methyl-4-isoxazolepropionic acid receptor
ApoE	Apolipoprotein E
APP	β-Amyloid precursor protein
APPL	Adaptor protein, phosphotyrosine interacting with PH domain and leucine zipper 1
BACE-1	β-APP-cleaving enzyme 1
BDNF	Brain-derived neurotrophic factor
β-CTF	Carboxy-terminal APP fragment generated by BACE-1
C9ORF72	Chromosome 9 open reading frame 72
CK	Casein kinase
CNS	Central nervous system
COPD	Chronic obstructive pulmonary disease
CSF	Cerebrospinal fluid
DLB	Dementia with Lewy bodies
DRG	Dorsal root ganglion
DS	Down syndrome
EEA	Early endosomal autoantigen
ERK	Extracellular signal-regulated kinase
FTD	Frontotemporal dementia
GAP	GTPase activating protein
GDI	GDP dissociation inhibitor
GEF	Guanine nucleotide exchange factor
GTPase	Guanosine triphosphatase
HAP40	Htt-associated protein 40
HD	Huntington’s disease
Htt	Huntingtin
hVPS45	Human Sec1p-like vacuolar protein sorting
IBD	Inflammatory bowel disease
IGF1	Insulin-like growth factor 1
IL-1β	Interleukin-1β
iPSC	Induced pluripotent stem cell
LTD	Long-term depression
LTP	Long-term potentiation
MAPK	Mitogen-activated protein kinase
MAPKAPK2	MAPK-activated protein kinase 2
MK2	MAPK-activated protein kinase 2
MKK	Mitogen-activated protein kinase kinase
MSK	Mitogen and stress-activated kinase
NGF	Nerve growth factor
NMDA	N-methyl-d-aspartate
PD	Parkinson’s disease
PH	Pleckstrin homology
PI3K	Phosphatidylinositol-3-kinase
RA	Rheumatoid arthritis
Rab5	Ras-related protein Rab5
SAPK	Stress-activated protein kinase
SNRI	Serotonin and norepinephrine reuptake inhibitor
SOD1	Superoxide dismutase 1
SORL1	Sortilin-related receptor 1
TNFα	Tumor necrosis factor α
